# Utility of combined CD39/CD73/CD38 expression for the detection of malignant T cells in Sézary syndrome

**DOI:** 10.1111/bjh.70562

**Published:** 2026-05-25

**Authors:** Sara Marchisio, Yuliya Yakymiv, Liyun Lin, Erika Ortolan, Verdiana Pullano, Renata Ponti, Fenna J. de Bie, Alita J. van der Sluijs‐Gelling, Willem H. Zoutman, Valentina Pala, Ferenc A. Scheeren, Maarten H. Vermeer, Ada Funaro, Pietro Quaglino, Gabriele Roccuzzo

**Affiliations:** ^1^ Laboratory of Immunogenetics, Department of Medical Sciences University of Turin Turin Italy; ^2^ Department of Neuroscience ‘Rita Levi Montalcini’ University of Turin Turin Italy; ^3^ Department of Medical Sciences, Dermatologic Clinic University of Turin Turin Italy; ^4^ Department of Dermatology Leiden University Medical Center Leiden the Netherlands; ^5^ Interdipartimental Center for Molecular Biotechnologies ‘Guido Tarone’ University of Turin Turin Italy

**Keywords:** CD38, CD39, CD73, flow cytometry, Sézary syndrome, tumour markers

## Abstract

Sézary syndrome (SS) is an aggressive cutaneous T‐cell lymphoma, characterized by erythroderma, lymphadenopathy and circulating malignant CD4^+^ T cells. Despite therapeutic advances, SS remains an incurable disease. Early diagnosis is therefore essential for timely therapeutic intervention; however, reliable biomarkers to identify circulating SS cells are still lacking. Recent studies highlighted dysregulation of the ectoenzymes CD39, CD73 and CD38 in SS, suggesting a potential role for these molecules in the disease. Thirty‐three patients were enrolled in this study, and CD39/CD73/CD38 expression was analysed on circulating CD4^+^ T cells by multiparametric flow cytometry, enabling discrimination between malignant and non‐malignant T‐cell subsets. SS cells exhibited marked CD39 or CD73 overexpression together with consistent CD38 downregulation, a pattern also confirmed in paired skin biopsy, whereas non‐malignant CD4^+^ T cells largely maintained expression profiles comparable to healthy controls. Genotyping further identified *ENTPD1* single nucleotide polymorphism rs10748643 as a contributor to CD39 dysregulation, defining a CD39‐permissive subgroup characterized by CD39^+^ SS cells. In conclusion, the combined overexpression of CD39 or CD73 and loss of CD38 defines a distinct tumour‐restricted immunophenotypic signature in SS. Integrating these ectoenzymes into flow cytometry panels may improve detection of malignant CD4^+^ T cells across blood and skin, eventually strengthening diagnostic accuracy.

## INTRODUCTION

Sézary syndrome (SS) is a rare and aggressive leukaemic variant of cutaneous T‐cell lymphoma (CTCL), defined by erythroderma, lymphadenopathies and neoplastic CD4^+^ T cells (Sézary cells) in skin, lymph node and peripheral blood.^1^ Despite therapeutic advances, SS remains incurable with low 5‐year overall survival rates.^2^, ^3^ Early diagnosis is therefore essential for timely therapeutic intervention. However, histopathological features lack disease specificity, leading to delayed and challenging diagnosis.^4^, ^5^ Accordingly, detection of circulating malignant T cells by flow cytometry (FC) represents essential diagnostic assessment.^1^, ^6^, ^7^

Phenotypically, SS cells are commonly defined by loss of CD26 and/or CD7 expression, together with additional tumour‐associated abnormalities, including diminished CD3, CD4 and CD2 expression.^8^ However, SS cells display marked heterogeneity.^9^ CD7 and/or CD26 loss is not observed in all patients, and similar phenotypic alterations may also occur in reactive non‐malignant CD4^+^ T cells, reducing disease specificity.^10^, ^11^

Additional markers such as KIR3DL2 (CD158k),^12^ chemokine receptor CCR4,^13^ immune checkpoint inhibitor PD‐1 (CD279)^10^ and TRBC1, used as a surrogate for T‐cell clonality,^14^ have improved diagnostic accuracy, yet retain relevant limitations.^15^, ^16^, ^17^ These markers have recently been incorporated into standardized multiparametric panels, including the one developed by the EuroFlow Consortium.^8^ However, their reliance on predominantly negative markers, combined with the phenotypic complexity of Sézary cells and partial overlap with non‐malignant subsets, still limits diagnostic robustness and precludes simplified analysis. Consequently, more reliable and disease‐specific markers are needed for standardized FC panels.

The adenosinergic pathway, comprising the ectonucleotidases CD39, CD73 and CD38, has emerged as a key immunoregulatory axis in cancer,^18^ yet its role in SS remains controversial.^19^ CD38 shows heterogeneous expression,^20^, ^21^, ^22^ whereas recent studies reported abnormal CD39 and CD73 levels in CD4^+^ T cells from SS patients, suggesting their implication in disease biology,^23^, ^24^, ^25^, ^26^ with emerging insights into CD39 regulation.^27^, ^28^ However, studies distinguishing CD39, CD73 and CD38 expression between malignant and benign CD4^+^ T‐cell subsets are limited, and analyses in cutaneous samples are scarce and largely restricted to immunohistochemistry.^20^, ^23^, ^25^ In addition, the contribution of genetic regulation to aberrant CD39 expression has not been fully elucidated. Collectively, these observations indicate that the adenosinergic axis remains underexplored but potentially relevant in SS, with important implications for understanding disease biology and identifying novel markers.

In this study, we used high‐dimensional FC to characterize CD39, CD73 and CD38 expression in circulating malignant and non‐malignant CD4^+^ T‐cell subsets from SS patients. We extended this analysis to paired skin and blood samples and investigated the impact of *ENTPD1/CD39* polymorphisms on CD39 regulation, aiming to identify reliable tumour‐restricted phenotypic markers to support the development of more robust FC panels for SS, improving diagnostic accuracy.

## MATERIALS AND METHODS

### Patients

This study included 27 patients with a confirmed SS diagnosis treated at the Dermatologic Clinic, Department of Medical Sciences, University of Turin, Città della Salute e della Scienza Hospital (Torino, Italy) between July 2020 and July 2025, independently of the disease course and treatment administered. Additionally, six SS patients from the Department of Dermatology, Leiden University Medical Center (Leiden, Netherlands) were included. Initial SS diagnosis was established according to the International Society for Cutaneous Lymphomas (ISCL), the United States Cutaneous Lymphoma Consortium (USCLC), and the European Organisation for Research and Treatment of Cancer (EORTC) guidelines.^6^ Table S1 summarizes the analysis performed for each SS patient, including transcript, genetic and FC analyses (Tables S2–S5). Further details are provided in Supporting Information.

### Flow cytometry

The experimental FC procedures and panels are illustrated in Tables S4 and S5. Conventional FC was performed on fresh peripheral blood samples from 27 SS patients and 28 healthy donors (HDs) using six‐colour FC panels (Panels 1–3). For spectral 22‐colour FC, cryopreserved peripheral blood mononuclear cells (PBMCs) from 21 SS, 10 HDs and 5 patients with benign erythrodermic dermatoses (EIDs) were stained with Panel 4. Figures S1 and S2 illustrate gating strategies for identification of malignant and non‐malignant CD4^+^ T cells. Panel 5 was used for matched PBMCs and fresh skin biopsy.

## RESULTS

### Patient characteristics

Thirty‐three patients with a confirmed SS diagnosis^6^ were included in the study (Table 1; Table S1). Overall, the cohort included 25 men and 8 women, with a median age of 70 years (range 46–86). At the time of inclusion, the median Sézary cell count was 2048 cells/mm^3^ (range 383–187 573), and the median CD4/CD8 ratio was 14 (range 0.74–177.88), reflecting the high tumour burden typically observed in SS. Eleven of these patients were newly diagnosed or untreated, whereas the remaining were patients on therapy.

**TABLE 1:** Clinical and disease‐related characteristics of SS patients (*n* = 33) at the time of study inclusion.

SS ID	Sex/age	Time from diagnosis (months)	Stage at study inclusion^a^	TCR‐Vβ family	SS cells/mm^3^	CD4:CD8	Systemic therapy
SS01	M/48	42	T4N3M0B2	Null	5148	108	ECP + BV
SS02	M/73	12	T4NxM0B2	13.1	1895	5.93	ECP
SS03	M/81	116	T2aNxM0B2	8	8203	15.44	Moga
SS04	M/70	14	T4NxM0B1	5.1	962	0.74	Moga
SS05	F/70	66	T4N1M0B1	22	429	4.08	ECP
SS06	M/62	85	T4NxM0B2	Null	4995	21.4	ECP
SS07	F/67	71	T4NxM0B2	13.6	5405	45.8	BV
SS08	F/76	1	T4N0M0B2	14	1371	7.21	ECP
SS09	M/70	11	T2bN0M0B2	Null	9026	25.69	ECP
SS10	M/83	46	T4N0M0B2	Null	1622	20.49	ECP + retinoids
SS11	M/81	31	T4NxM0B2	22	1423	13.16	Retinoids
SS12	M/74	1	T4NxM0B2	16	5062	20.23	Untreated
SS14	F/49	1	T4NxM0B2	20	7093	59.61	ECP
SS15	M/68	0	T2NxM0B2	2	4406	12.83	Untreated
SS17	M/83	6	T4NxM0B2	Null	1070	2.6	ECP
SS18	M/86	2	T4NxM0B1	Null	305	1.8	ECP + s. ster.
SS19	M/79	3	T4NxM0B2	5.1	11 976	33.61	ECP
SS20	M/81	12	T4NxM0B1	Null	801	16.57	ECP
SS22	M/74	0	T4NxM0B1	Null	906	12.16	Untreated
SS23	F/61	1	T4NxM0B2	22	2499	8.08	Untreated
SS25	M/69	1	T4NxM0B1	13.6	946	15.78	Untreated
SS27	M/77	17	T4NxM0B2	Null	1163	4.24	Untreated
SS28	M/80	1	T4N0M0B1	Null	935	1.65	Untreated
SS30	M/67	1	T4N0M0B2	2	2172	14	MTX
SS31	M/46	3	T2N1M0B1	13.1	383	5.13	Untreated
SS32	M/65	3	T2N0M0B2	17	1826	12.77	s. ster.
SS34	M/73	0	T4NxM0B2	Null	19 653	31.3	Untreated
SS35	M/68	65	T4N2M0B2	Null	7680	77.79	s. ster.
SS36	F/60	7	T4NxM0B2	8	N/A	25.05	s. PUVA
SS37	M/77	0	T4NxM0B2	7.2	2880	177.88	Chemotherapy + s. ster.
SS38	F/59	2	T4N3M0B2	22	187 573	113.88	s. ster.
SS39	M/79	1	T4NxMxB2	7.2	3020	3.82	Untreated
SS40	F/64	0	T4NxM0B2	5.1	1924	8.89	Untreated

Abbreviations: BV, brentuximab‐vedotin; ECP, extracorporeal photopheresis; F, female; M, male; Moga, mogamulizumab; MTX, methotrexate; s. PUVA, systemic psoralene UVA light therapy; s. ster., systemic steroids; SS, Sézary syndrome; TCR, T cell receptor.

^a^ Sézary syndrome TNMB staging system at study inclusion.^6^

### High‐dimensional profiling reveals tumour‐restricted CD39/CD73 overexpression and CD38 reduction in SS patients

CD4^+^ T cells from 27/33 SS patients were immunophenotyped with six‐colour FC. Compared with 28 HDs, data confirmed the overexpression of CD39 or CD73 in specific patient subgroups, both in terms of cell frequency and median fluorescence intensity (MFI) (Figure S3), with transcript analysis showing concordant mRNA increases (Figure S4). Additional analysis revealed a marked reduction in CD38 levels in patients' CD4^+^ T cells compared with HDs (Figure S3). Overall, these findings confirmed the distinct immunophenotypic profile of CD4^+^ T cells in SS patients, characterized by CD39 and/or CD73 overexpression and consistent CD38 decrease compared to HDs.

**FIGURE 1:**
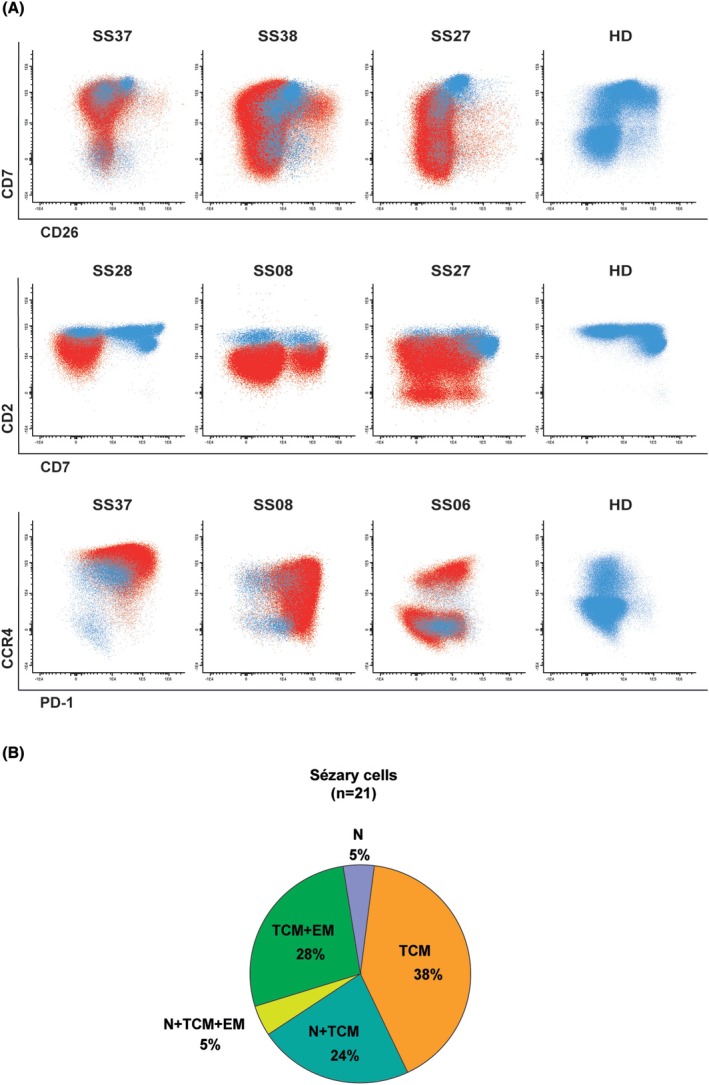
Phenotypic heterogeneity of Sézary cells. (A) Representative dot plots showing inter‐ and intrapatient heterogeneity of tumour cells. SS cells (red) and non‐malignant CD4^+^ T cells (blue) are shown for three representative SS patients compared with CD4^+^ T cells from one representative HD. Patterns of expression of CD26 versus CD7 (upper panel), CD7 versus CD2 (central panel), PD‐1 versus CCR4 (lower panel) are illustrated. (B) Distribution of maturation phenotypes among tumour Sézary cells in the cohort (*n* = 21). EM, effector memory; HD, healthy donor; N, naïve; SS, Sézary syndrome; TCM, transitional/central memory; TE, terminal effector.

To further characterize the malignant CD4^+^ T cells, we performed high‐dimensional 22‐colour FC in 21/33 SS patients (including 15 patients previously analysed by 6‐colour FC), 5 EIDs and 10 HDs. SS cells displayed marked inter‐ and intra‐patient heterogeneity (Figure 1A). As previously described,^10^, ^29^ SS cells revealed a generalized reduction in canonical T‐cell marker expression, including CD45, CD3, CD4, CD2, CD26 and CD7, compared with CD4^+^ T cells from HDs. Although uncommon, increased expression of some of these markers was detected in a minority of cases (Table S6), underscoring the pronounced phenotypic variability of SS cells. In addition, most individuals showed increased PD‐1 and CCR4 expression on SS cells. However, patients' non‐malignant CD4^+^ T cells also exhibited a predominant shift towards higher CCR4 and a modest increase in PD‐1 levels compared with HDs (Table S6). Furthermore, SS cells often displayed multiple coexisting differentiation stages (Figure 1B; Figure S5), predominantly clustering within early stages, including transitional/central memory and naïve, whereas terminal effector phenotypes were not detected, in line with previous studies.^30^, ^31^

Since non‐malignant CD4^+^ T cells in SS may overlap with the malignant phenotype, as reflected by the CCR4 and PD‐1 skewing observed in our cohort, we compared CD39, CD73 and CD38 expression across the two subsets to determine whether ectoenzyme dysregulations were tumour‐restricted.

**FIGURE 2:**
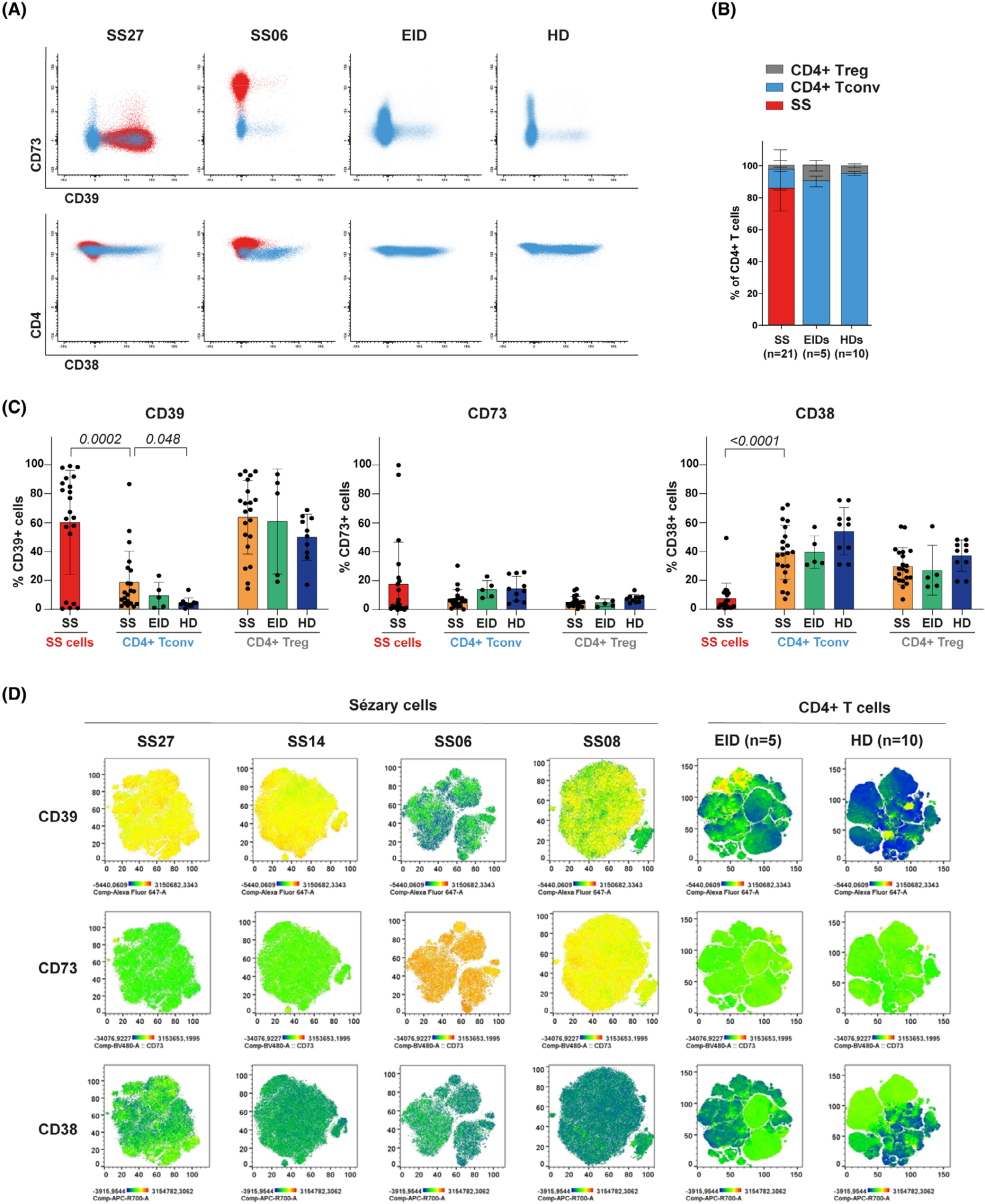
Ectoenzymes expression in malignant and non‐malignant CD4^+^ T lymphocytes. (A) Flow cytometry dot plots showing CD39, CD73 and CD38 expression in malignant and non‐malignant CD4^+^ T cells from two representative patients, one EID and one HD. (B) Distribution of SS cells, Tconv and Treg within CD4^+^ T cells across groups. (C) Percentages of CD39^+^, CD73^+^ and CD38^+^ cells within malignant, Tconv and Treg cells across groups. (D) t‐SNE plots showing CD39, CD73 and CD38 distribution in malignant cells from two CD39^+^ patients (SS27, SS14), one CD73^+^ (SS06) and one CD39^+^CD73^+^ (SS08) patient, compared with CD4^+^ T‐cell concatenated data from EIDs and HDs. EIDs, erythrodermic dermatoses; HD, healthy donor; SS, Sézary syndrome; Tconv, conventional T; Treg, regulatory T.

In patients showing CD39 or CD73 overexpression on CD4^+^ T cells (Figure S3), CD39^+^ and CD73^+^ subsets almost exclusively overlapped with the malignant compartment, whereas their expression levels on non‐malignant CD4^+^ T cells were comparable to EIDs and HDs. CD38 showed the opposite pattern, being consistently reduced in SS cells relative to HDs (Figure 2A). Next, non‐malignant CD4^+^ T cells were further divided into regulatory T (Treg) and conventional T (Tconv) cells. As expected, the malignant clone dominated the CD4^+^ pool in patients, reducing the relative contribution of both Treg and Tconv populations (Figure 2B). Comparative analysis showed that SS cells exhibited significantly higher CD39 frequencies compared with autologous Tconv cells, whereas only minor changes were detected in Tconv cells between SS patients and HDs. Conversely, Treg cells maintained constitutively high CD39 expression across all groups, but their low frequency in SS patients made their overall contribution to CD4^+^ T cells negligible relative to malignant cells (Figure 2B,C). Similarly, aberrant CD73 expression, when present, was restricted to SS cells, with no upregulation in non‐malignant subsets. Parallel analysis in SS patients revealed the predominance of CD38‐low malignant cells, independent of their CD39 or CD73 expression, with non‐malignant CD4^+^ T cells maintaining CD38 levels comparable to controls (Figure 2C). Finally, t‐distributed stochastic neighbour embedding (t‐SNE) analysis confirmed a uniform overexpression of CD39 or CD73, with some patients positive for both markers, and a homogeneous downregulation of CD38 across malignant SS cells compared with controls (Figure 2D). Collectively, these data demonstrate that CD39 and CD73 overexpression, together with reduced CD38, constitutes a tumour‐specific signature of SS cells, with minimal overlap with non‐malignant CD4^+^ T cells.

### Comparable CD39/CD73 expression profiles in circulating and skin‐derived malignant T cells

**FIGURE 3:**
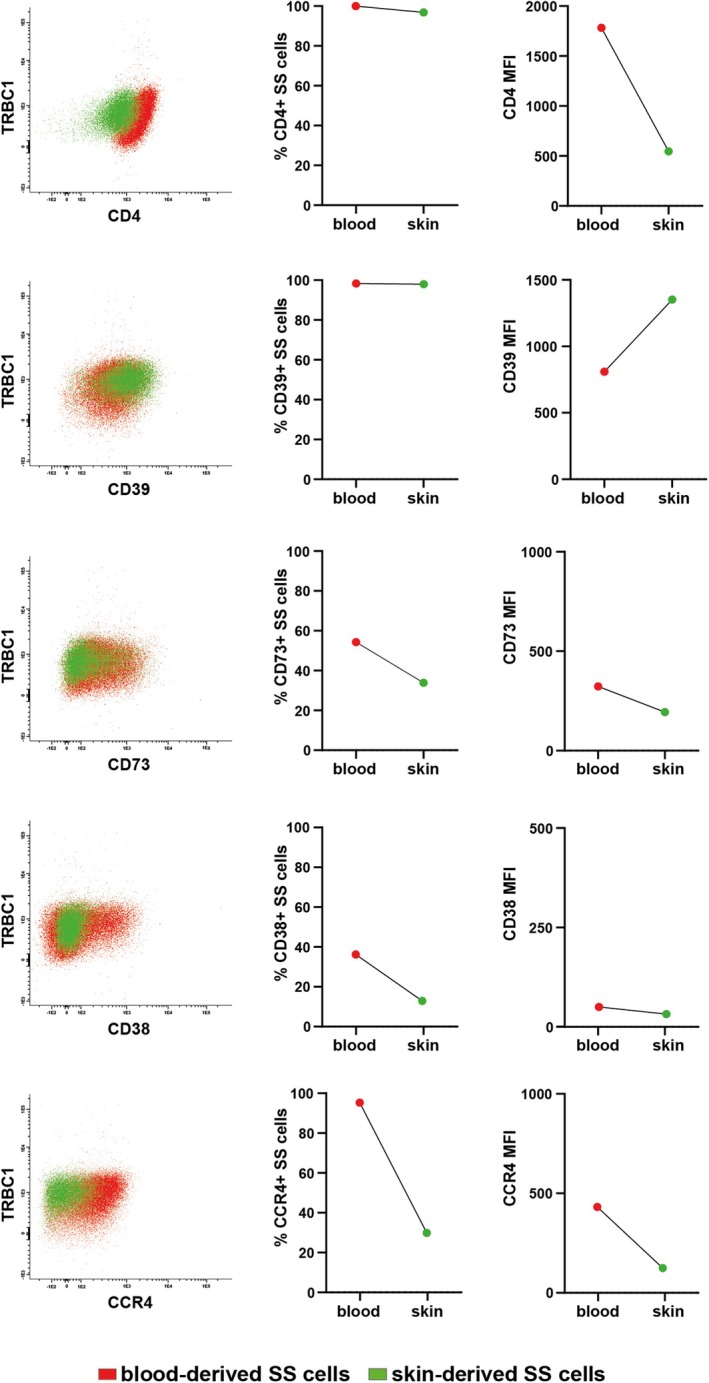
Comparison of blood‐ and skin‐derived malignant CD4+ T cells in SS34. Flow cytometry dot plots (left panels) of blood‐derived and skin‐derived SS cells from matched samples of patient SS34 for CD4, CD39, CD73, CD38 and CCR4 expression. Corresponding cell frequencies (middle panels) and MFI values (left panels) are shown for each marker. Red and green dots indicate paired blood‐ and skin‐derived SS cells respectively. MFI, median fluorescence intensity; SS, Sézary syndrome.

To characterize phenotypic differences between circulating and skin‐resident malignant T cells, we performed FC on paired peripheral blood and skin samples from one SS patient, evaluating CD39, CD73 and CD38 expression, as well as skin‐homing molecule CCR4 on SS cells (Figure 3).

CD4 expression was markedly higher on circulating tumour cells compared with skin‐derived counterparts. Circulating malignant cells were predominantly CD39^+^ (99.2%) with intermediate CD73 expression (54.3%). While the frequency of CD39^+^ cells was similar across compartments, skin‐resident cells showed higher CD39 MFI and slightly lower CD73 frequency and intensity compared to blood. CD38 displayed intermediate expression in blood whereas it was nearly absent in the skin, with consistently low MFI across compartments. Finally, CCR4 expression was strongly diminished in skin‐resident malignant cells compared to circulating ones, consistent with previous evidence.^11^ While showing compartment‐specific modulation of CD38 and CCR4, the malignant clone retains its CD39/CD73 phenotype across blood and skin, highlighting their potential utility as tumour‐associated markers in identifying circulating and skin‐derived malignant cells.

### *ENTPD1* rs10748643 genotype regulates CD39 expression in Sézary cells

To explore the mechanisms underlying the interindividual variability in CD39 expression on SS cells, we analysed the *ENTPD1*/*CD39* genomic region, focusing on two single nucleotide polymorphisms (SNPs), rs7096317 (G/A) and rs10748643 (A/G), both previously associated with the regulation of CD39 expression in healthy T cells.^32^, ^33^, ^34^, ^35^ In silico analysis showed complete linkage disequilibrium between the two variants (*r* ^2^ = 1; *D*′ = 1) (Figure S6), as confirmed by genotyping data from 25/33 SS patients (Table S7). Ranking these SNPs based on predicted regulatory activity by the FORGEdb database,^36^ rs10748643 displayed the highest regulatory score (Figure S6b) and was therefore selected for downstream analyses.

**FIGURE 4:**
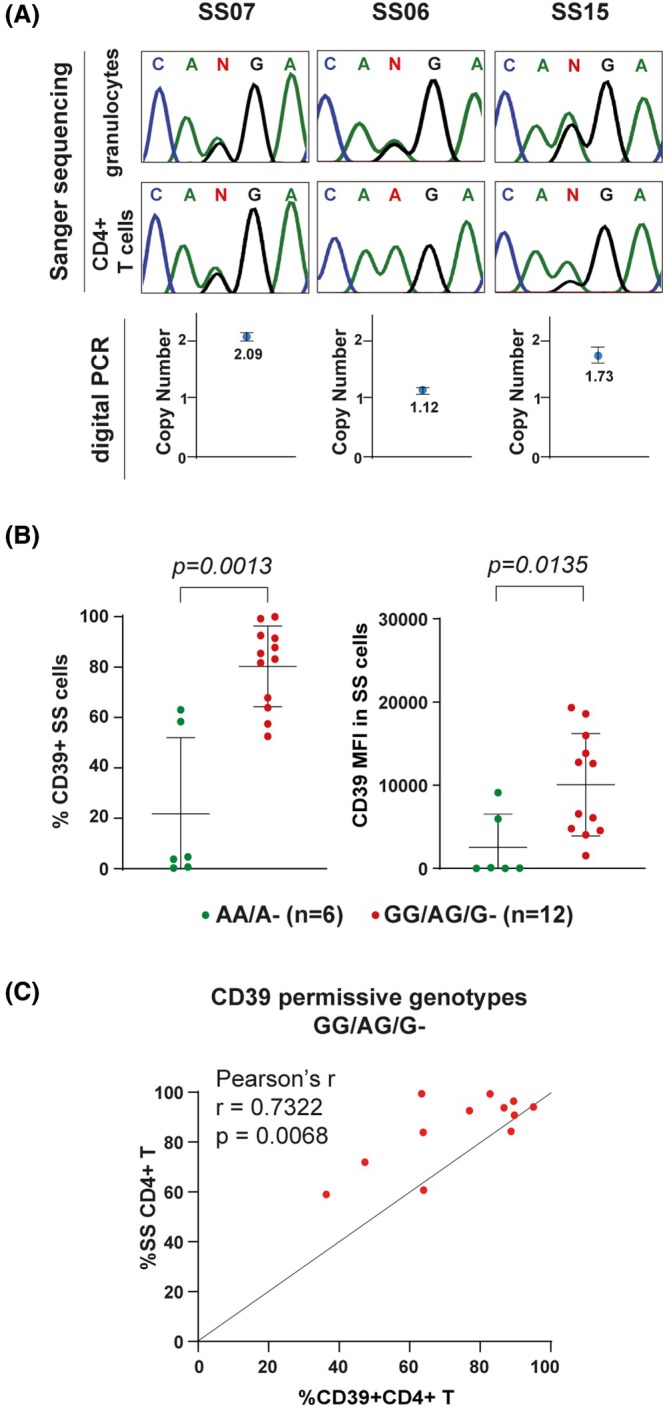
*ENTPD1* rs10748643 genotyping and CD39 expression in SS patients. (A) Upper panels show Sanger sequence electropherograms showing rs10748643 genotype in granulocytes (germline reference) and CD4^+^ T cells from a representative A/G (SS07), A/− (SS06) and an A/G patient with reduced G‐allele signal (SS15) in CD4^+^ T cells. Lower panels show the corresponding copy number data obtained by digital PCR (WT = 2; LOSS <2). (B) Percentages of CD39^+^ SS cells (left) and CD39 MFI (right) in AA/A− and GG/AG/G− patients. (C) Correlation plot between the frequency of CD39^+^CD4^+^ T cells (*X*‐axis) and percentage of SS cells (*Y*‐axis) within CD4^+^ T cells in patients with permissive genotypes (GG/AG/G−). MFI, median fluorescence intensity; SS, Sézary syndrome.

As an association between rs10748643 and CD39 expression in SS was previously reported,^28^ we sought to validate these findings in our cohort by integrating Sanger genotyping with *ENTPD1* copy number analysis. This is particularly relevant given the high genomic instability of Sézary cells at chromosome 10q.^37^, ^38^ Digital PCR performed in 14/25 patients revealed that the *ENTPD1* region was diploid in six cases, whereas allelic loss was detected in eight cases (Table 2). Among the latter, SS06 showed complete G‐allele loss, providing evidence of loss of heterozygosity. While SS15 exhibited a less pronounced G‐allele reduction relative to the AG germline genotype observed in granulocytes, likely reflecting subclonal allelic loss within the malignant T cell given the patient's high tumour burden (Figure 4A).

**TABLE 2:** ENTPD1 copy number status, SNP rs10748643 genotype and corresponding CD39 expression in SS patients (*n* = 19).

SS ID	Copy number	Genotype rs10748643	CD39+/SS cells (%)	CD39‐AF647 MFI in SS cells
SS02	WT	G/G	63.75	4027
SS06	LOSS	A/−	0.30	29
SS07	WT	A/G	85.32	12 594
SS08	LOSS	A/−	58.27	9105
SS09	WT	G/G	91.33	13 826
SS10	N/A	G/G	81.53	15 978
SS12	WT	A/A	3.81	3
SS14	WT	A/G	99.76	18 576
SS15	LOSS^a^	A/G^a^	77.39	17 491
SS19	N/A	A/A	4.70	4
SS20	WT	A/G	67.70	6552
SS23	N/A	A/G	52.45	1512
SS25	N/A	A/G	87.60	6083
SS27	LOSS	G/−	99.00	19 320
SS28	N/A	G/G	57.36	4781
SS35	LOSS	G/−	92.35	12 761
SS36	LOSS	A/−	62.98	5939
SS37	LOSS	A/−	0.67	65
SS38	LOSS	G/−	83.03	4541

Abbreviations: AF647, Alexa fluor 647; MFI, median fluorescence intensity; SNP, single nucleotide polymorphism; SS, Sézary syndrome; WT, wild type.

^a^ A/G patient with reduced G‐allele signal (excluded from subsequent subgroup analyses).

When stratified by rs10748643 genotype, patients carrying at least one G allele (G/G, A/G or G/−) displayed a significantly higher percentage of CD39^+^ SS cells compared with A/A or A/− patients (80.1 ± 16.00% vs. 21.79 ± 30.17%) and higher MFI values (10 046 ± 6158 vs. 2524 ± 3999, respectively) (Figure 4B; Table 2), thereby defining G‐carrying genotypes as ‘permissive’ for CD39 upregulation. Within this subgroup, a strong positive correlation was observed between the frequency of CD39^+^CD4^+^ T cells and the proportion of SS cells (Pearson's *r* = 0.7322, *p* = 0.0068) (Figure 4C). Survival curves did not suggest a different prognosis among the two groups (A−/AA vs. GG/AG/G−) (Figure S7; Table S7); however, the limited number of patients precludes definitive conclusions. Overall, these findings extend previous observations on rs10748643 role in modulating CD39 expression in Sézary cells and further support CD39 as a reliable biomarker for malignant cell identification in patients with CD39‐permissive genotypes.

## DISCUSSION

The lack of tumour‐specific markers for Sézary cell identification represents a major diagnostic challenge in SS.^8^ In this study, we evaluated the expression of the ectoenzymes CD39, CD73 and CD38, recently implicated in CTCL biology,^19^ to explore their potential utility as phenotypic SS markers.

FC analysis confirmed abnormal CD39 and CD73 expression on circulating CD4^+^ T cells in our cohort. The distribution of these phenotypic subsets was consistent with previous findings, with CD39^+^ subjects representing the predominant population while only a minority were CD73^+^, including few CD39^+^CD73^+^ patients.^23^, ^24^ Noteworthy, transcript analysis supported CD39 and CD73 bona fide cellular overexpression rather than alternative mechanisms reported in other biological settings.^39^, ^40^

Deeper immunophenotypic characterization showed that aberrant CD39 and CD73 expression was largely restricted to Sézary cells, rather than reflecting a global modulation across non‐malignant CD4^+^ subsets. Although CD39 levels were modestly increased in CD4^+^ Tconv cells from SS patients compared with controls, this increase was minimal compared with the marked overexpression observed on malignant cells, reinforcing a predominantly tumour‐restricted pattern. These findings partially align with Sonigo et al.,^24^ who reported increased CD39 in non‐malignant T cells, though their analysis did not discriminate between Tconv and Treg subsets. By separating these populations, our data revealed that patients' Treg cells, physiologically exhibiting high CD39 levels,^41^ were comparable to controls; therefore, minimally contributing to the overall CD39 overexpression on CD4^+^ cells in patients. To our knowledge, this is the first study to systematically profile CD39 and CD73 expression across both malignant and non‐malignant CD4^+^ T‐cell subsets in SS, minimizing subset‐driven confounders.

In parallel, CD38 expression was markedly reduced on total CD4^+^ T cells from patients, reflecting the predominance of malignant SS cells lacking this marker, as non‐malignant counterparts retained expression levels similar to controls. This observation is consistent with prior studies describing low/intermediate CD38 expression in SS^10^, ^20^, ^21^, ^22^ and supports its utility as a negative discriminator between tumour and normal CD4^+^ lymphocytes, complementary to CD39 and CD73 for SS cell identification.

In line with previous evidence,^16^ non‐malignant CD4^+^ T cells showed increased CCR4 expression and a modest rise in PD‐1 compared with HDs, further reducing the discriminatory power of these markers in identifying SS cells and strengthening the need for reliable diagnostic markers.

Because Sézary cells disseminate between blood and skin, understanding whether their immunophenotype remains stable across compartments is essential for interpreting how blood‐based biomarkers reflect the biology of skin‐resident disease. To date, no FC studies have directly examined CD39 or CD73 expression on skin‐infiltrating Sézary cells. Previous reports suggested similar expression of these markers in blood and skin in CTCL but relied on assessment performed with different methodologies and at different time points, limiting direct comparison and interpretability.^23^, ^25^ A recent study highlighted CD39 as a promising diagnostic marker in SS, combining blood FC with skin lesions transcriptomics and proteomics; however, direct FC characterization of skin‐infiltrating malignant cells remains unexplored.^42^ Likewise, CD38 FC assessment in skin of SS patients is still lacking. Our matched analysis provides the first direct FC comparison of CD39, CD73 and CD38 between blood and skin in SS, although caution is warranted since these observations are based on a single patient. Despite preserved TRBC1 clonality, compartment‐specific differences emerged. CD39 levels were substantially higher in skin‐resident malignant cells, whereas CD73 was modestly reduced, suggesting that the cutaneous microenvironment may actively shape ectonucleotidase activity while preserving the underlying tumour‐specific signature,^43^, ^44^ supporting their use for identifying malignant cells in both blood and skin.

While our FC analysis revealed lower CD38 levels in skin compared with circulating SS cells, single‐cell RNA sequencing results from Ta et al.^20^ reported high CD38 expression in skin‐infiltrating malignant cells from aggressive CTCL subtypes, including SS with large cell transformation, supporting its negative prognostic impact. These differences likely reflect variation in disease context, aggressiveness and analytical approach, underscoring the need for direct phenotypic profiling of skin‐derived malignant cells to clarify CD38 clinical relevance and its utility for patient risk stratification. Importantly, the potential impact of skin dissociation procedures on CD39, CD73 and CD38 surface detection should be ruled out, and additional larger studies are required to validate these compartment‐specific patterns.

Beyond ectoenzyme expression, CCR4, consistently expressed by circulating malignant cells, was markedly reduced in skin‐resident clone, consistent with previous observations,^11^ and may explain the lower cutaneous response relative to blood in patients treated with mogamulizumab, an anti‐CCR4 monoclonal antibody.^45^

In our cohort, SS phenotypic heterogeneity extended to CD39 expression, with only a subset of patients being CD39^+^. To investigate this variability, we examined genetic determinants of *ENTPD1* regulation. Consistent with Picozza et al.,^28^ rs10748643 modulated CD39 expression in our cohort. Moreover, patients carrying at least one G allele showed a strong correlation between CD39^+^CD4^+^ T cells and tumour burden, further supporting *ENTPD1* genotyping as a straightforward stratification tool to identify patients in whom CD39 can serve as a reliable tumour marker. Few studies explored the prognostic role of rs10748643 in SS, reporting conflicting results^28^, ^46^; our data showed no clear prognostic impact; however, limited cohort size precludes definitive conclusions. Importantly, digital PCR can reveal allelic *ENTPD1* loss or imbalance in SS patients; hence, integrating Sanger sequencing with copy number variant analysis can uncover subclonal genomic alterations^47^ that might be missed by conventional sequencing approaches.

While rs10748643 represents a relevant determinant, as supported by recent evidence showing its role in modulating NFIC transcription factor binding affinity in healthy T and natural killer (NK) cells,^48^ it is unlikely to be the only driver of CD39 dysregulation in SS. Additional regulatory mechanisms may act in concert, including transcriptional regulators such as the JAK/STAT pathway,^49^ and epigenetic modulation, which is gaining increasing attention in CTCL.^50^, ^51^ Preliminary analyses from our group revealed no association between *ENTPD1* promoter methylation and CD39 expression (data not shown), suggesting DNA methylation does not have a major regulatory role in this context. Therefore, additional regulatory mechanisms, including those controlling CD73 expression, warrant further investigation.

Methodological FC variabilities across studies complicate direct clinical implementation and underline the need for standardized protocols.^10^ Moreover, therapeutic pressure can drive clonal selection and phenotypic shifts in SS,^9^ underscoring the importance of demonstrating marker stability over time,^52^ particularly for monitoring tumour burden and response to depleting therapies such as mogamulizumab.

CD39, CD73 and CD38 should not be interpreted as stand‐alone markers, but rather within a multiparametric panel including established SS‐associated markers such as CD26, CD7, CCR4 and TRBC1, which together improve specificity and help distinguish SS from overlapping entities such as adult T‐cell leukaemia/lymphoma, in which CD39 overexpression has also been reported.^53^ Although multicentre validation is still required, our findings support the integration of these markers into FC diagnostic panels as robust biomarkers with strong potential for SS cell detection. Beyond their diagnostic value, these ectoenzymes play a central role in shaping the immunosuppressive milieu characteristic of SS,^23^ highlighting the therapeutic promise of targeting the adenosinergic pathway in this disease.^19^ Moreover, integrating CD39 genotyping may further help in the identification of individuals most likely to benefit from CD39‐based diagnostic or therapeutic approaches.

In a setting where reliable SS markers remain an unmet need, these insights pave the way for the integration of these markers into diagnostic FC panels enabling more accurate SS cell identification.

## AUTHOR CONTRIBUTIONS

**Sara Marchisio:** Conceptualization; data curation; formal analysis; investigation; methodology; visualization; writing – original draft; writing – review and editing. **Yuliya Yakymiv:** Conceptualization; methodology; data curation; formal analysis; investigation; visualization; writing – original draft; writing – review and editing. **Liyun Lin:** Investigation; writing – review and editing. **Erika Ortolan:** Conceptualization; data curation; formal analysis; investigation; methodology; writing – review and editing. **Verdiana Pullano:** Methodology; writing – review and editing. **Renata Ponti:** Resources; writing – review and editing. **Fenna J. de Bie:** Methodology; writing – review and editing. **Alita J. van der Sluijs‐Gelling:** Methodology; writing – review and editing. **Willem H. Zoutman:** Methodology; writing – review and editing. **Valentina Pala:** Writing – review and editing. **Ferenc A. Scheeren:** Supervision; writing – review and editing. **Maarten H. Vermeer:** Supervision; funding acquisition; writing – review and editing. **Ada Funaro:** Conceptualization; data curation; funding acquisition; supervision; writing – review and editing. **Pietro Quaglino:** Conceptualization; funding acquisition; supervision; writing – review and editing. **Gabriele Roccuzzo:** Formal analysis; resources; writing – review and editing.

## FUNDING INFORMATION

This research was funded by the Research Projects of National Relevant Interest (Prot.20209KY3Y7 to P.Q.), IMMUNO‐CANCER‐PRIN‐2021 (to P.Q.) and KWF Dutch Cancer Society (to M.H.V.).

## CONFLICT OF INTEREST STATEMENT

The authors declare no conflict of interest.

## ETHICS STATEMENT

The data were collected in accordance with the Declaration of Helsinki, and the study was approved by the local Institutional Review Board (Comitato Etico Interaziendale A.O.U. Città della Salute e della Scienza di Torino‐A.O. Ordine Mauriziano‐A.S.L. TO1, Italy) and Medical Ethics Review Committee of the Leiden University Medical Center (Netherlands). Full written informed consent was obtained from all patients involved in the study.

## Supporting information


**Figure S1.** Gating strategy for the identification of malignant and non‐malignant CD4+ T cells in SS patients.
**Figure S2.** Sézary cells and CD4+ T‐cell gating strategies.
**Figure S3.** CD39, CD73 and CD38 expression in CD4^+^ T lymphocytes from SS patients (*n* = 27) and HDs (*n* = 28).
**Figure S4.** CD39 and CD73 transcript levels.
**Figure S5.** Maturation phenotypes of malignant SS cells.
**Figure S6.** Genomic position and regulatory annotation of rs7096317 and rs10748643 within the *ENTPD1* locus.
**Figure S7.** Kaplan–Meier overall survival curves for patients stratified by rs10748643 SNP genotype (*n* = 20).
**Table S1.** Overview of analyses performed in SS patients.
**Table S2.** Detailed primer information for real time PCR (CD39, CD73 and TBP) and ENTPD1 SNP genotyping (rs10748643 and 7096317).
**Table S3.** Detailed primer and probe information for ddPCR.
**Table S4.** Summary of flow cytometry panels and experimental procedures.
**Table S5.** Flow cytometry panels and reagents' information.
**Table S6.** Individual values of the median fluorescence intensity (MFI) of malignant SS cells and mean ± SD of non‐malignant CD4^+^ T cell from SS patients (*n* = 21) and CD4^+^ T cells from HDs (*n* = 10) for the indicated markers.
**Table S7.** Genotype distribution of rs10748643 and rs7096317 in our SS cohort (*n* = 25), and patients analysed for survival analysis.

## Data Availability

The collected data are not publicly available to protect patients' privacy and comply with ethical requirements. Aggregated data supporting the study findings are available from the corresponding author upon a reasonable request.
