# Supplementary material for: Utility of combined CD39/CD73/CD38 expression for the detection of malignant T cells in Sézary syndrome
Source: Br J Haematol. 2026 May 25;209(1):42–53. doi: 10.1111/bjh.70562 (PMC13340541; doi:10.1111/bjh.70562)
Supplement: Supplementary file 1 — Figure S1. Gating strategy for the identification of malignant and non‐malignant CD4+ T cells in SS patients. Figure S2. Sézary cells and CD4+ T‐cell gating strategies. Figure S3. CD39, CD73 and CD38 expression in CD4+ T lymphocytes from SS patients (n = 27) and HDs (n = 28). Figure S4. CD39 and CD73 transcript levels. Figure S5. Maturation phenotypes of malignant SS cells. Figure S6. Genomic position and regulatory annotation of rs7096317 and rs10748643 within the ENTPD1 locus. Figure S7. Kaplan–Meier overall survival curves for patients stratified by rs10748643 SNP genotype (n = 20). Table S1. Overview of analyses performed in SS patients. Table S2. Detailed primer information for real time PCR (CD39, CD73 and TBP) and ENTPD1 SNP genotyping (rs10748643 and 7096317). Table S3. Detailed primer and probe information for ddPCR. Table S4. Summary of flow cytometry panels and experimental procedures. Table S5. Flow cytometry panels and reagents' information. Table S6. Individual values of the median fluorescence intensity (MFI) of malignant SS cells and mean ± SD of non‐malignant CD4+ T cell from SS patients (n = 21) and CD4+ T cells from HDs (n = 10) for the indicated markers. Table S7. Genotype distribution of rs10748643 and rs7096317 in our SS cohort (n = 25), and patients analysed for survival analysis. [file BJH-209-42-s001.docx]

**Supplementary Materials and methods**

**Patients and samples collection**

Initial SS diagnosis was established according to ISCL/USCLC/EORTC guidelines^1,2^. All patients had a skin examination and TCR gene rearrangement analysis using the Euroclonality/BIOMED-2 system^3^. Blood involvement was defined by the immunophenotypic detection of a circulating T-cell clone, assessed either by TCR-vβ repertoire analysis (IOTest Beta Mark TCR Vβ Repertoire Kit, Beckman Coulter) or by TRBC1 monotypic expression^4,5^, together with aberrant CD4^+^ T-cell immunophenotype consistent with SS (CD26- and/or CD7-). Clinical and disease-related data, including absolute tumor cell counts and TNMB disease stage at study inclusion, were retrospectively obtained from medical records.

***Blood samples***

EDTA-anticoagulated blood samples were collected, and peripheral blood mononuclear cells (PBMCs) were isolated from fresh blood samples by Ficoll-Histopaque density gradient (Sigma Aldrich) and subsequently cryopreserved. When indicated, CD4^+^ T cells were negatively isolated using magnetic beads CD4^+^ T Cell Isolation Kit (Miltenyi Biotec, 30-096-533). In addition, granulocytes were isolated from blood samples following Ficoll separation and blood cell lysis, and their DNA was extracted as reference genomic DNA.

***Skin samples***

Fresh skin biopsies were collected and dissociated immediately for flow cytometry analysis using the human Whole Skin Dissociation kit according to the manufacturer’s instructions (Miltenyi Biotec, 130-101-540). Briefly, after scalpel dissection the biopsy was placed in cold RPMI medium in a Petri dish, minced, and transferred to a gentleMACS C tube containing the enzyme mix (excluding Enzyme P), incubated for 2h at 37°C, transferred to a gentleMACS Octo Dissociator (Miltenyi Biotec, 130-093-235) and run using the program h_skin_01. After, cells were spin down at 300g for 1 min, resuspended in RMPI medium, passed through a 70 µm strainer and washed with 1 ml of RPMI medium. After centrifugation, cells were resuspended in 100ul of PBS and stained according to the immunostaining protocol.

**Flow cytometry (FC) staining and analysis**

Overview of the analysed experimental settings (acquisition and analysis) and panels are shown in Table S4-S5. Conventional FC was performed on fresh peripheral blood samples (Panels 1-3), data were acquired on a FACSCanto cytometer (BD Biosciences) and analysed with FlowJo software v10.6 (BD Biosciences). For the spectral 22-colour FC analysis, cryopreserved PBMC were stained with Panel 4. Data were acquired on a Cytek Aurora 3-laser cytometer (Cytek Biosciences) and analysed using Infinicyt software (Cytognos SL) and FlowJo software v10.6. SS cells were identified based on aberrant expression of SS-related markers (e.g. monotypic TRBC1) or atypical lineage features (e.g. CD3^dim^ or CD4^dim^), whereas non-malignant CD4^+^ T cells were defined using a subtractive gating approach, excluding SS cells and displaying biphasic TRBC expression pattern (Figure S1). Among tumor cells, naïve (N), transitional/central memory (TCM), effector memory (EM) and terminal effector memory (TE) cells were defined based on the combined expression of CD27, CD45RA, and CD62L^6^ (Figure S2a). Regulatory T (Treg; CD25^high^CD127^low^) and conventional T (Tconv) cells were defined within the non-malignant CD4^+^ T cell subset (Figure S2b). Matched cryopreserved PBMCs and fresh skin samples were stained with Panel 5, and data were acquired on a FACS Celesta cytometer (BD Biosciences).

**t-distributed stochastic neighbour embedding (t-SNE) analysis**

t-SNE dimensionality reduction was performed on panel 4 (Table S5) to visualize the global distribution of tumor SS cells. For each patient, SS cells were first identified on Infinicyt (Cytognos SL), and the corresponding populations were exported for dimensionality reduction. t-SNE was generated in FlowJo v10.6 software (BD Biosciences) excluding CD39, CD73, and CD38, which were subsequently overlaid as independent variables for visualization. To generate a reference control map, data from 10 concatenated HDs, as well as 5 EIDs, were combined into a single file and analysed in parallel with patient samples. The same scale and settings were applied across all analyses to allow direct comparison between SS patients and controls.

**Gene expression analysis by real-time PCR**

For gene expression analysis, isolated CD4^+^ T cells from freshly collected PBMCs were incubated for 5 hours with 0.2 mg/ml Proteinase K (Zymo Research, D3001-2-5) in DNA/RNA Shield 1X (Zymo Research, R1100) to improve the digestion efficiency. Total RNA was extracted using the Direct-Zol RNA MiniPrep (Zymo Research, R2052), and reverse-transcribed into cDNA with the RevertAid First Strand cDNA Synthesis Kit (ThermoFisher, K1662), according to manufacturer's protocol. Real-time quantitative PCR was performed on a CFX96 Thermal Cycler (BioRad) using iTaq Universal SYBR Green SuperMix (BioRad, 172-5121). Each reaction contained 10 ng of template cDNA and 500nM of each primer (Table S2). Ct values for the genes of interest (CD39 and CD73) were normalized to the housekeeping gene TBP and relative gene expression was calculated using the 2⁻^ΔΔCt^ method^7^ compared with HDs levels.

**Sanger sequencing**

Sanger sequencing was performed on genomic DNA extracted from isolated CD4^+^ T cells, or PBMCs (when tumor cells were ≥ 70% of the PBMCs) and matched granulocyte DNA, the latter representing the reference germline compartment. DNA was extracted using the ReliaPrep Blood gDNA Miniprep System (Promega, A5081), according to manufacturer’s instructions, and quantified with a NanoDrop2000 spectrophotometer (ThermoFisher Scientific). Target regions within *ENTPD1* rs10748643 and rs7096317 were amplified by PCR. PCR reactions (25 µL) contained KAPA2G Fast HotStart polymerase (Kapa Biosystems, KK5512), 10 µM primers (Table S2), 10 mM dNTPs, and 1 µL of DNA. The PCR program included an initial denaturation (95°C, 3 min), a touchdown phase of 14 cycles (95°C, 15 s; 60–50°C, 15 s; 72°C, 3 s), and 25 stable cycles (95°C, 15 s; 50°C, 15 s; 72°C, 3 s). PCR products were visualized on 1% agarose gel using the ChemiDoc™ XRS+ System (BioRad) and purified using ExoSAP (ThermoFisher Scientific) and FastAP (ThermoFisher Scientific) in a 1:2 ratio (15 min at 37°C, 15 min at 85°C). Sanger sequencing was performed by Eurofins Genomics using the forward primers. Chromatograms were analyzed using ChromasPro software (Technelysium), and genotypes were compared with CD39 expression profiles obtained by flow cytometry.

**Droplet digital PCR (ddPCR)**

Copy number status of *ENTPD1* (10q24.1), including SNP rs10748643, was determined on genomic DNA extracted from CD4^+^ T cells, or PBMC (when tumor cells were ≥ 50% of the PBMCs). The copy number of the *ENTPD1* was calculated relative to *DNM3* (1q24.3); a reference locus, generally copy number stable in Sézary Syndrome patients^8^. Each ddPCR reaction (22µl) contained 15-25 ng of DNA, ddPCR Supermix for Probes (No dUTP) (Bio-Rad Laboratories), FAM- and HEX-labelled hydrolysis probes, forward and reverse primers (Table S3), and DNA restriction digestion enzyme HaeIII (New England Biolabs). All components were added directly to the dPCR reaction solution, as previously described^9^. Droplets were generated using an AutoDG System (Bio-Rad) and PCR amplification was performed in a T100 Thermal Cycler (Bio-Rad). Cycle parameters were as follows: enzyme activation for 10 minutes at 95⁰C; denaturation for 30 seconds at 94⁰C; annealing and extension for 1 minute at 60⁰C for 40 cycles; enzyme deactivation for 10 minutes at 98⁰C; cool step for 30 minutes at 4⁰C followed by an infinite cooling at 12⁰C. The ramp rate for all cycles was 2⁰C/second. Droplets were measured by a QX200 Droplet Reader (Bio-Rad) and analyzed using Quantasoft software (version 1.7.4; Bio-Rad).

For robust quantification, a minimum of 10,000 acceptable droplets was required per reaction, and data from duplicates were merged. Copy number and associated 95% confidence intervals (CI) were determined by QuantaSoft according to Poisson distribution on scored droplets. A diploid genotype (WT) was defined when the copy number 95% CI encompasses the value 2. Allelic imbalance, by means of a loss of ENTPD1, was defined when the upper limit of the 95% CI was below 2. Because copy number was assessed on bulk DNA from CD4⁺ T cells or PBMCs, values were interpreted in the context of tumor burden.

**Statistical analysis**

Statistical analyses were performed using GraphPad Prism v7. 0 (GraphPad Software). For two-group comparisons unpaired parametric Mann-Whitney or non-parametric Welch’s t-tests were used, according to data distribution and variance. Multiple comparisons of CD39, CD73, and CD38 expression across CD4^+^ T-cell subsets within SS patients (tumor, Tconv, Treg) were analysed using paired non-parametric Friedman test (one-way ANOVA). For analyses between independent groups (SS, EIDs, HDs), multiple comparisons were performed separately for T_conv_ and T_reg_ using unpaired non-parametric Kruskal–Wallis test (one-way ANOVA). Correlations between CD39^+^CD4^+^ T cell frequency and tumor cell burden variables were assessed using Pearson’s correlation coefficient (r). Survival curves were generated based on the Kaplan-Meier method and analysed through the Log-rank test; Overall survival (OS) was measured from the SS diagnosis until death. Data for patients who remained alive were censored at the date of their last visit with the medical team. A p-value < 0.05 was considered statistically significant. If not otherwise indicated, data are shown as mean ± SD.

**REFERENCES**

1 Olsen E, Vonderheid E, Pimpinelli N, *et al.* Revisions to the staging and classification of mycosis fungoides and Sézary syndrome: a proposal of the International Society for Cutaneous Lymphomas (ISCL) and the cutaneous lymphoma task force of the European Organization of Research and Treatment of Cancer (EORTC). *Blood* 2007; 110:1713–22.

2 Olsen EA, Whittaker S, Willemze R, *et al.* Primary cutaneous lymphoma: recommendations for clinical trial design and staging update from the ISCL, USCLC, and EORTC. *Blood* 2022; 140:419–37.

3 Langerak AW, Groenen PJTA, Brüggemann M, *et al.* EuroClonality/BIOMED-2 guidelines for interpretation and reporting of Ig/TCR clonality testing in suspected lymphoproliferations. Leukemia. 2012; 26:2159–71.

4 Gibson JF, Huang J, Liu KJ, *et al.* Cutaneous T-cell lymphoma (CTCL): Current practices in blood assessment and the utility of T-cell receptor (TCR)-Vβ chain restriction. *J Am Acad Dermatol* 2016; 74:870–7.

5 Horna P, Shi M, Olteanu H, Johansson U. Emerging Role of T-cell Receptor Constant β Chain-1 (TRBC1) Expression in the Flow Cytometric Diagnosis of T-cell Malignancies. *Int J Mol Sci* 2021; 22:1817.

6 Botafogo V, Pérez-Andres M, Jara-Acevedo M, *et al.* Age Distribution of Multiple Functionally Relevant Subsets of CD4+ T Cells in Human Blood Using a Standardized and Validated 14-Color EuroFlow Immune Monitoring Tube. *Front Immunol* 2020; 11. doi:10.3389/fimmu.2020.00166.

7 Pfaffl MW. A new mathematical model for relative quantification in real-time RT-PCR. *Nucleic Acids Res* 2001; 29:45e–45.

8 Zoutman WH, Nell RJ, Versluis M, *et al.* Accurate Quantification of T Cells by Measuring Loss of Germline T-Cell Receptor Loci with Generic Single Duplex Droplet Digital PCR Assays. *The Journal of Molecular Diagnostics* 2017; 19:236–43.

9 Zoutman WH, Nell RJ, van der Velden PA. Usage of Droplet Digital PCR (ddPCR) Assays for T Cell Quantification in Cancer. , 2019; 1–14.

**Supplementary Figures**

***
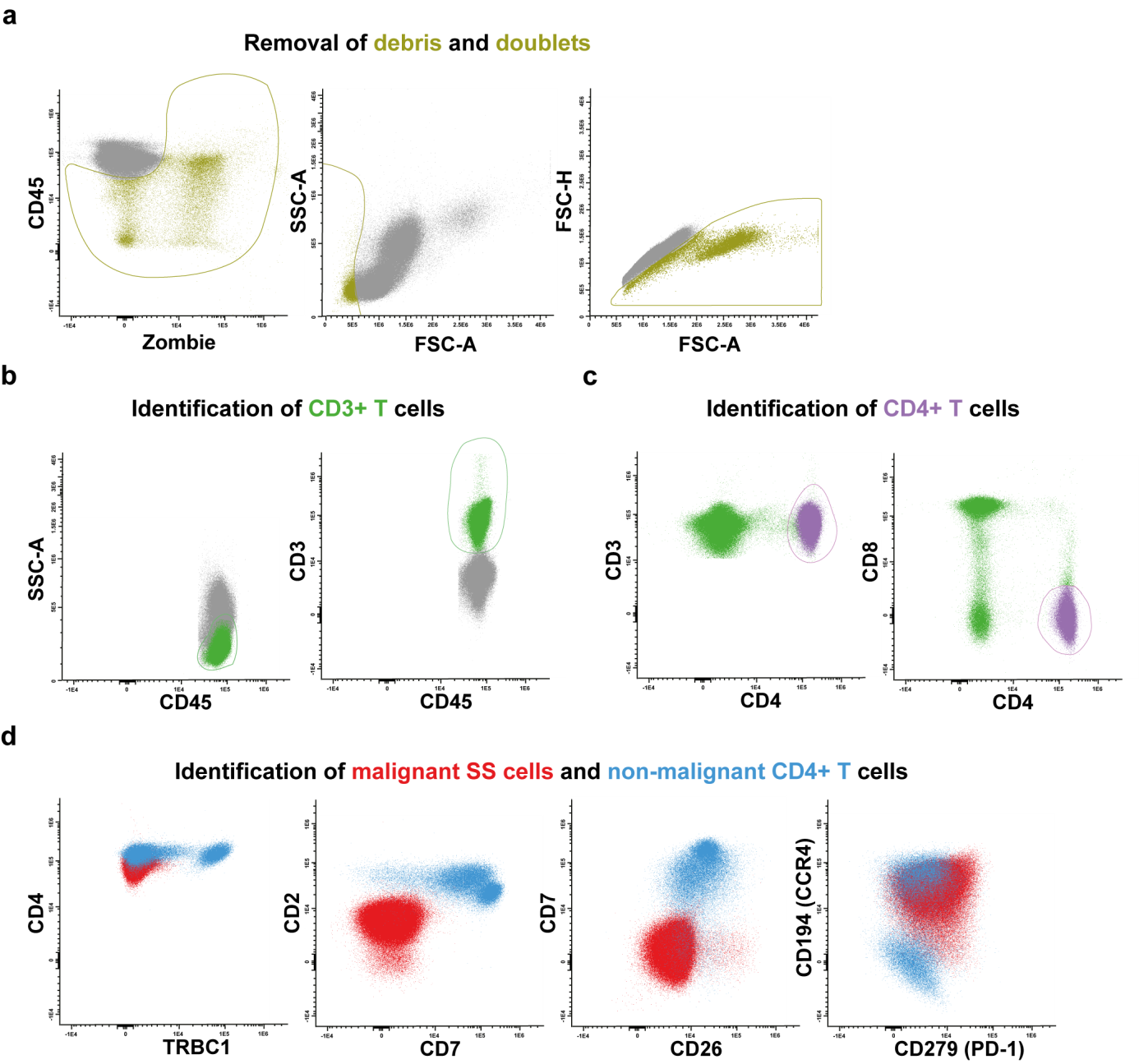
***

**Figure S1: Gating strategy for the identification of malignant and non-malignant CD4+ T cells in SS patients.** Gating hierarchy from a representative PBMC sample: (**a**) exclusion of debris (dark yellow; Zombie^+^ and CD45^-^, low FSC-A events) and doublets (dark yellow; events outside the diagonal in the FSC-H vs FSC-A plot); (**b**) identification of CD3^+^ T cells (green) as CD45^+^CD3^+^ events; (**c**) identification of CD4^+^ T lymphocytes (pink) as CD3^+^CD4^+^ and CD8^-^CD4^+^ events; (**d**) identification of malignant SS cells (red) and non-malignant CD4^+^ T cells (blue) based on the combination of SS-related and lineage markers.


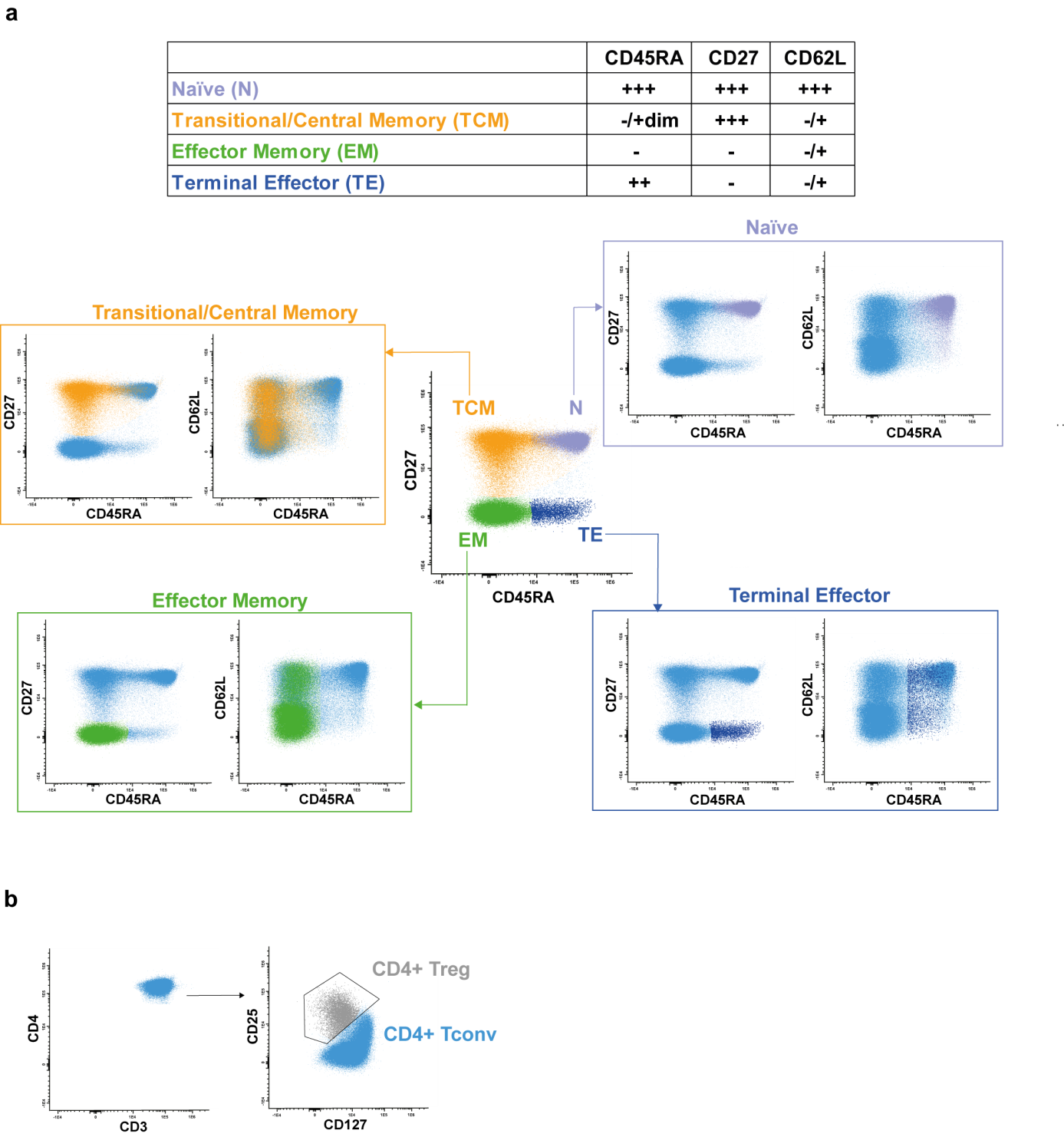


**Figure S2: Sézary cells and CD4+ T cell gating strategies. a.** Gating strategy for maturation profile CD4^+^ T-cell subsets. In the upper panel, schematic overview of marker combination used to define naïve (N; CD45RA^+++^, CD27^+++^, CD62L^+++^), transitional/central memory (TCM; CD45RA^-/+dim^, CD27^+++^, CD62L^-/+^), effector memory (EM; CD45RA^-^, CD27^-^, CD62L^-/+^) and terminal effector (TE; CD45RA^++^, CD27^-^, CD62L^-/+^) CD4^+^ T cell subsets. The lower panel shows the stepwise gating strategy applied to a representative HD to identify each subset. **b.** Regulatory T cells gating strategy. Flow cytometry dot plots showing gating strategy for identification of CD4^+^ regulatory T cells Treg (grey; CD127^low^/CD25^high^) and conventional T cells Tconv (blue) within non-malignant CD4^+^ T cells.

***
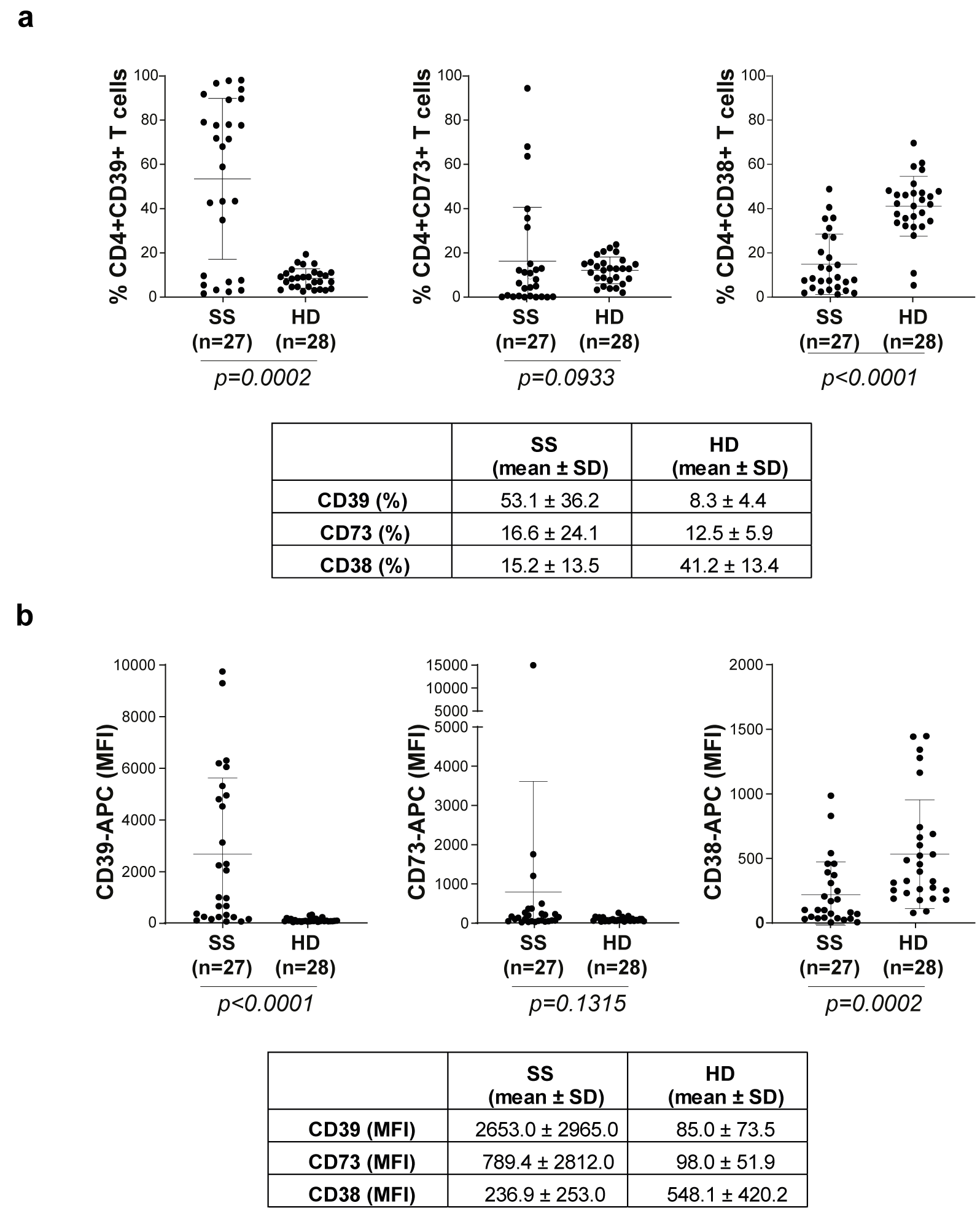
***

**Figure S3: CD39, CD73 and CD38 expression in CD4^+^ T lymphocytes from SS patients (n=27) and HDs (n=28). (a)** Percentages of CD39^+^ (left panel), CD73^+^ (central panel) and CD38^+^ (right panel) cells and (**b**) corresponding median fluorescence intensity (MFI) levels within CD4^+^ T cells from SS patients and HDs. Each dot represents a single individual. Horizontal bars indicate the mean ± SD. At the bottom of each panel, tables illustrating the mean ± SD of (**a**) percentages and (**b**) MFI values of CD39, CD73 and CD38 within CD4^+^ T cells from SS and HDs.


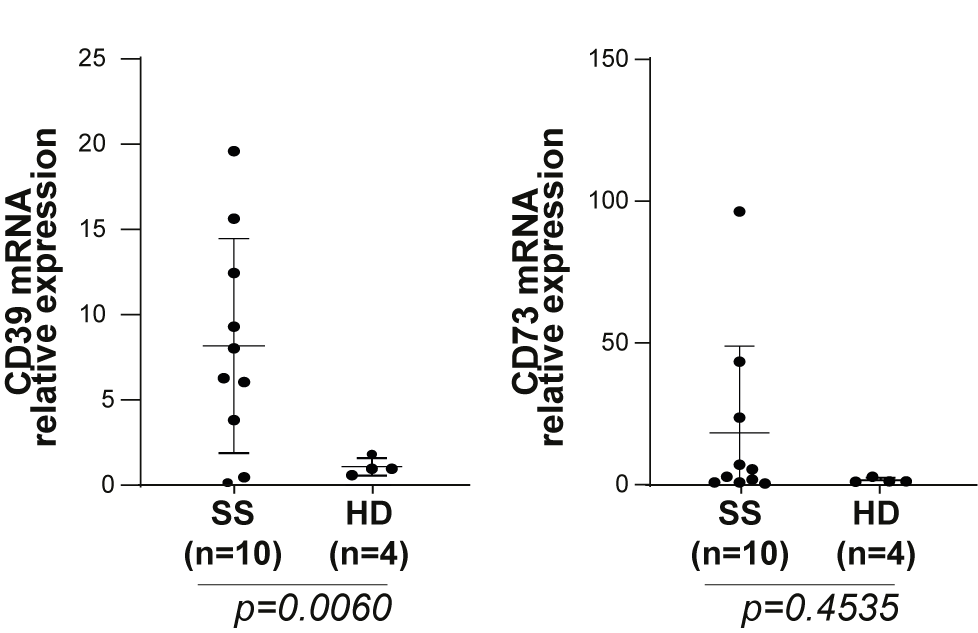


**Figure S4: CD39 and CD73 transcript levels.** Relative mRNA levels of CD39 (left panel) and CD73 (right panel) in CD4^+^ T cells from SS patients and HDs. Data are expressed as mean ± SD relative to TBP transcripts.


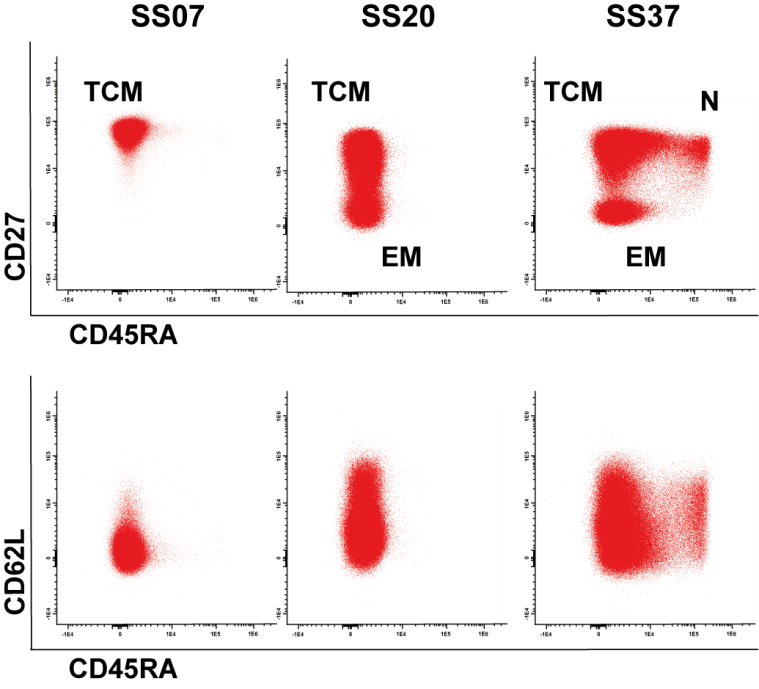


**Figure S5: Maturation phenotypes of malignant SS cells.** Representative dot plots from three SS patients displaying distinct maturation profiles: TCM (SS07), TCM+EM (SS20), and N+TCM+EM (SS37). Abbreviations: N, naïve; TCM, transitional/central memory; EM, effector memory; TE, terminal effector memory.


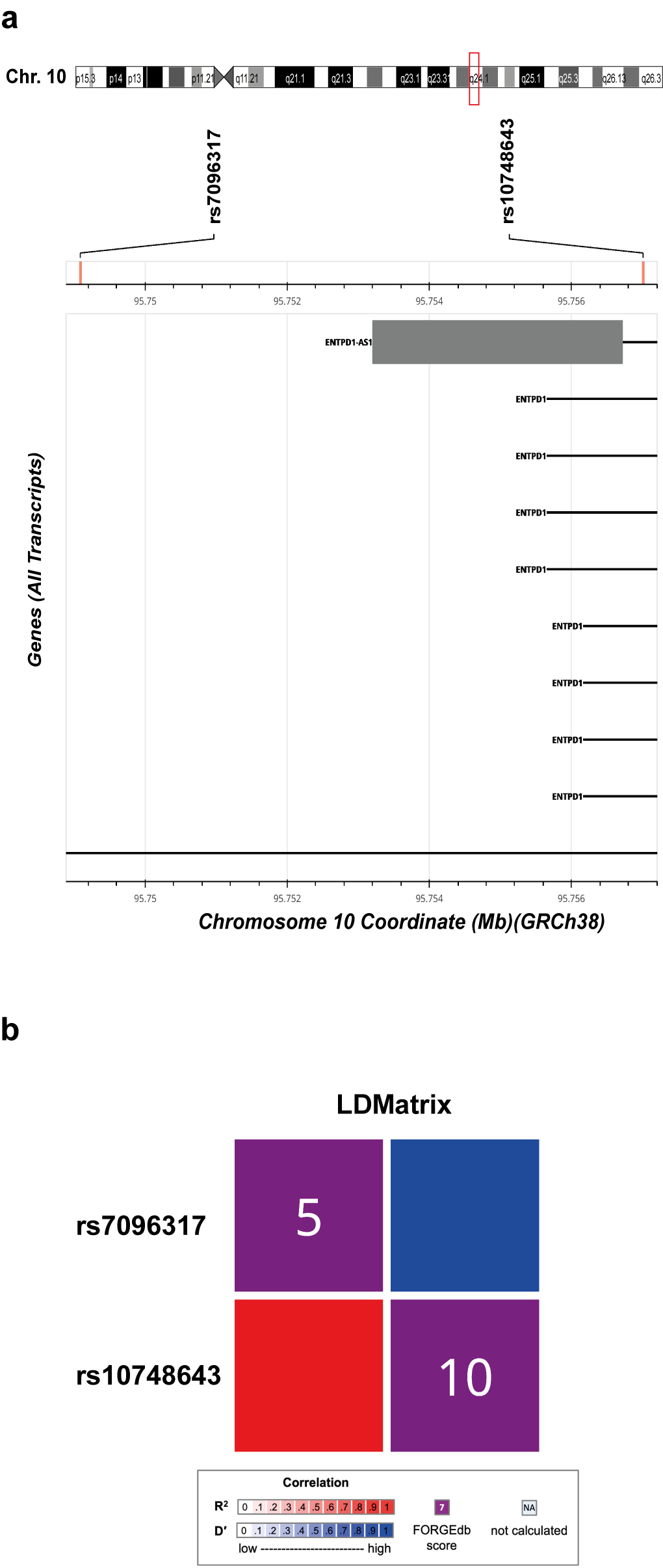


**Figure S6: Genomic position and regulatory annotation of rs7096317 and rs10748643 within the *ENTPD1* locus. (a)** Schematic representation of the *ENTPD1* locus on chromosome 10 (cytoband 10q24.1) showing the genomic positions of the two SNPs analysed, rs7096317 (chr10:95.774908; G/A) and rs10748643 (chr10:95.757007; A/G), based on the GRCh38 reference assembly. Positions are indicated in megabases (Mb). **(b)** Linkage disequilibrium (LD) matrix showing the pairwise LD values (r²=1 and D′=1) between the two SNPs, together with their corresponding FORGEdb regulatory scores. rs10748643 displays the maximum FORGEdb (score 10), indicating a higher predicted regulatory impact compared with rs7096317 (score 5). Genomic coordinates and LD metrics were obtained using LDlink (https://ldlink.nih.gov; Human GRCh38.p14).


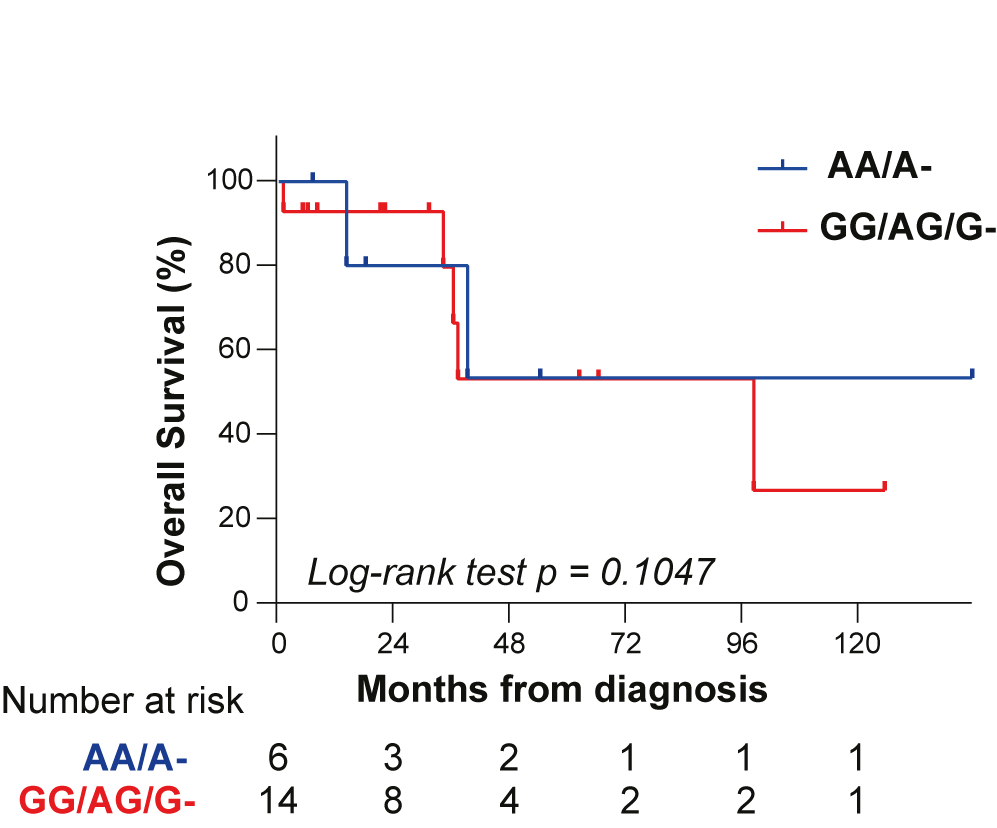


**Figure S7: Kaplan-Meier overall survival curves for patients stratified by rs10748643 SNP genotype (n=20).** Comparison of overall survival between SS patients carrying the AA/A- genotype and those with the GG/AG/G- genotype.

**Supplementary Tables**

| SS ID | Conventional FC (6-colour) | Spectral FC  (22-colour) | Skin FC  (12-colour) | Real-time PCR | Genotyping |
| --- | --- | --- | --- | --- | --- |
| SS01 | Y |  |  |  |  |
| SS02 | Y | Y |  |  | Y |
| SS03 | Y |  |  |  |  |
| SS04 | Y |  |  |  |  |
| SS05 | Y |  |  |  |  |
| SS06 | Y | Y |  | Y | Y |
| SS07 | Y | Y |  | Y | Y |
| SS08 | Y | Y |  |  | Y |
| SS09 | Y | Y |  |  | Y |
| SS10 | Y | Y |  |  | Y |
| SS11 | Y |  |  |  |  |
| SS12 | Y | Y |  |  | Y |
| SS14 | Y | Y |  |  | Y |
| SS15 | Y | Y |  |  | Y |
| SS17 | Y |  |  |  |  |
| SS18 | Y |  |  |  | Y |
| SS19 | Y | Y |  |  | Y |
| SS20 | Y | Y |  | Y | Y |
| SS22 | Y |  |  | Y | Y |
| SS23 | Y | Y |  |  | Y |
| SS25 | Y | Y |  | Y | Y |
| SS27 | Y | Y |  | Y | Y |
| SS28 | Y | Y |  |  | Y |
| SS30 | Y |  |  | Y | Y |
| SS31 | Y |  |  | Y | Y |
| SS32 | Y |  |  | Y | Y |
| SS34 | Y |  | Y | Y | Y |
| SS35 |  | Y |  |  | Y |
| SS36 |  | Y |  |  | Y |
| SS37 |  | Y |  |  | Y |
| SS38 |  | Y |  |  | Y |
| SS39 |  | Y |  |  |  |
| SS40 |  | Y |  |  |  |

**Table S1: Overview of analyses performed in SS patients.**

Abbreviations: FC, flow cytometry; Y, yes.

| Real time PCR | | |
| --- | --- | --- |
|  | **Forward primer**  **(5’ to 3’)** | **Reverse primer**  **(5’ to 3’)** |
| CD39 | AGGTGCCTATGGCTGGATTAC | CCAAAGCTCCAAAGGTTTCCT |
| CD73 | CAGTACCAGGGCACTATCTGG | AGTGGCCCCTTTGCTTTAAT |
| TBP | CGGCTGTTTAACTTCGCTTC | CACACGCCAAGAAACAGTGA |
| *ENTPD1* Sanger sequencing | | |
|  | **Forward primer**  **(5’ to 3’)** | **Reverse primer**  **(5’ to 3’)** |
| rs10748643 | GTAGAGGGAGGAAATAG | TGGCTACTCATGCTAT |
| rs7096317 | TTGGGAAGACAGCTTCAGAGG | TTTGCTGCACAAAATGTACCC |

**Table S2: Detailed primer information for real time PCR (CD39, CD73 and TBP) and ENTPD1 SNP genotyping (rs10748643 and 7096317).**

| Assay | Description | Forward primer  (5’ to 3’) | Reverse primer  (5’ to 3’) | Probe  (5’ to 3’) | Label |
| --- | --- | --- | --- | --- | --- |
| ENTPD1 | Target | TCTTTTGGGCATTTTCTGCC | ACCCTATACTCCCTCACTGC | GCCAGCTGCTTGCCAGCGTGT | FAM |
| DNM3 | Reference | CTAAACACCTCTGCTGATTTCTGC | CCGCCTTTCATGATGCCAATG | TGAGCCACCCCTTGCGAATCACCT | HEX |

**Table S3: Detailed primer and probe information for ddPCR.**

|  | Conventional FC (6-colour) | | | Spectral FC (22-colour) | Skin FC |
| --- | --- | --- | --- | --- | --- |
|  | **Panel 1** | **Panel 2** | **Panel 3** | **Panel 4** | **Panel 5** |
| Sample | Fresh PB | Fresh PB | Fresh PB | Cryopreserved PBMCs | Fresh Skin Biopsies  Cryopreserved PBMCs |
| Acquisition | BD FACS Canto (BD Biosciences) | BD FACS Canto (BD Biosciences) | BD FACS Canto (BD Biosciences) | Cytek Aurora 3-lasers (Cytek Biosciences) | BD FACS Celesta (BD Biosciences) |
| Analysis | FlowJo (BD Biosciences) | FlowJo (BD Biosciences) | FlowJo (BD Biosciences) | Infinyct (Cytognos SL) FlowJo (BD Biosciences) | Infinyct (Cytognos SL) FlowJo (BD Biosciences) |

**Table S4: Summary of flow cytometry panels and experimental procedures.**

Abbreviations: FC, flow cytometry; PB, peripheral blood.

| Panel 1 | | | | |
| --- | --- | --- | --- | --- |
| Marker | **Fluorochrome** | **Clone** | **Source** | **Catalogue number** |
| CD39 | FITC | A1 | eBioscience | 11-0399-42 |
| CD26 | PE | L272 | BD | 340423 |
| CD8 | PerCPCy5.5 | RPA-T8 | eBioscience | 45-0088-42 |
| CD19 | PECy7 | HIB19 | eBioscience | 25-0199-42 |
| CD73 | APC | AD2 | eBioscience | 12-0739-42 |
| CD4 | APC-H7 | RPA-T4 | BD | 560158 |
| Panel 2 | | | | |
| Marker | **Fluorochrome** | **Clone** | **Source** | **Catalogue number** |
| CD3 | FITC | UCHT1 | Biolegend | 300440 |
| CD25 | PE | BC96 | eBioscience | 12-0259-42 |
| CD127 | PerCPCy5.5 | eBioRDR5 | eBioscience | 45-1278-42 |
| CD194 (CCR4) | PeCy7 | L291H4 | Biolegend | 359410 |
| CD39 | APC | A1 | eBioscience | 17-0399-42 |
| CD4 | APC-H7 | RPA-T4 | BD | 560158 |
| Panel 3 | | | | |
| Marker | **Fluorochrome** | **Clone** | **Source** | **Catalogue number** |
| CD4 | FITC | RPA-T4 | eBioscience | 11-0049-42 |
| CD279 (PD-1) | PE | MIH4 | BD | 557946 |
| CD8 | PerCPCy5.5 | RPA-T8 | eBioscience | 45-0088-42 |
| CD39 | PeCy7 | A1 | eBioscience | 25-0399-42 |
| CD38 | APC | HIT2 | eBioscience | 17-0389-42 |
| CD3 | APC-H7 | SK7 | BD | 560176 |
| Panel 4 | | | | |
| Marker | **Fluorochrome** | **Clone** | **Source** | **Catalogue number** |
| CD27 | BV421 | M-T271 | BD | 562513 |
| CD2 | PacB | TS1/8 | BD | 644485 |
| CD73 | BV480 | AD2 | BD | 746568 |
| CD45RA | BV510 | HI100 | BD | 563031 |
| CD4 | BV605 | SK3 | BD | 566908 |
| CD62-L | BV650 | DREG56 | Biolegend | 304832 |
| CD127 | BV711 | HIL-7R-M21 | BD | 563165 |
| CD26 | BV750 | L272 | BD | 747167 |
| CD3 | BV786 | SK7 | BD | 563800 |
| TRBC1 | FITC | JOVI-1 | Immunostep | JOVIF |
| CD45 | AF532 | HI30 | eBioscience | 58-0459-42 |
| CD183 | PE | 1C6/CXCR3 | BD | 550633 |
| CD196 | PE CF594 | 11A9 | BD | 564816 |
| CD25 | PeCy5 | BC96 | Biolegend | 302607 |
| CCR10 | PerCPCy5.5 | 1B5 | BD | 564772 |
| CD7 | RB705 | 4H9 | BD | 757979 |
| CD279 (PD-1) | RB744 | EH12.1 | BD | 570478 |
| CD194 (CCR4) | PeCy7 | L291H4 | Biolegend | 359410 |
| CD39 | AF647 | A1 | BD | 567505 |
| CD38 | APC-R700 | HIT2 | BD | 564980 |
| Zombie NIR |  |  | Biolegend | 423105 |
| CD8 | APC-H7 | SK1 | BD | 641400 |
| Panel 5 | | | | |
| Marker | **Fluorochrome** | **Clone** | **Source** | **Catalogue number** |
| TRBC1 | BV421 | JOVI-1 | BD | 747979 |
| CD45 | BV480 | HI30 | BD | 566115 |
| CD38 | BV605 | HIT2 | BD | 562444 |
| CD73 | BV650 | AD2 | BD | 742633 |
| CD4 | BV786 | SK3 | BD | 563877 |
| CD194 (CCR4) | PE | L291H4 | Biolegend | 359412 |
| CD39 | PE CF594 | A1 | BD | 567668 |
| CD7 | APC | M-T701 | BD | 561604 |
| CD8 | R718 | SK1 | BD | 567354 |
| CD26 | RB705 | L272 | BD | 757729 |
| FVS780 | APC-H7 |  | BD | 565388 |
| CD3 | RB545 | UCHT1 | BD | 569197 |

**Table S5: Flow cytometry panels and reagents' information.**

Abbreviations: AF532, Alexa fluor 532; AF647, Alexa fluor 647; APC, Allophycocyanin; APCH7, Allophycocyanin-Hilite 7; APC-R700, Allophycocyanin-R700; BV, Brilliant Violet; FITC, Fluorescein isothiocyanate; FVS780, Fixable Viability Stain 780; NIR, Near-infrared; PacB, Pacific Blue; PE, Phycoerythrin; PE-Cy7, Phycoerythrin-Cyanin 7; PerCPCy5.5, Peridinin-chlorophyll protein-complex cyanin 5; RB, RealBlue.

| Malignant SS cells (SS patients=21) | | | | | | | | |
| --- | --- | --- | --- | --- | --- | --- | --- | --- |
|  | **CD45**  **BV480** | **CD3**  **RB545** | **CD4**  **BV786** | **CD2**  **PacB** | **CD26**  **BV750** | **CD7**  **RB705** | **PD-1**  **RB744** | **CCR4**  **PE** |
| SS02 | 44132 | 116376 | 108991 | 9352 | 6189 | 78792 | 4424 | 34030 |
| SS06 | 95207 | 39713 | 247391 | 39493 | 8815 | 20986 | 2889 | 1624 |
| SS07 | 45304 | 101752 | 84425 | 6557 | 5025 | 1545 | 5876 | 30286 |
| SS08 | 63705 | 70774 | 109987 | 8333 | 6602 | 3673 | 43060 | 19079 |
| SS09 | 37169 | 27796 | 76082 | 2617 | 6011 | 1202 | 3927 | 14907 |
| SS10 | 47389 | 63552 | 87043 | 23015 | 7116 | 3022 | 1865 | 16161 |
| SS12 | 58804 | 39960 | 124252 | 16978 | 5794 | 41278 | 26611 | 16188 |
| SS14 | 46591 | 33582 | 108340 | 7407 | 4686 | 2864 | 12645 | 22421 |
| SS15 | 52611 | 41010 | 148416 | 17538 | 4740 | 2016 | 12272 | 189519 |
| SS19 | 40746 | 85134 | 113763 | 18355 | 5909 | 3876 | 11548 | 4453 |
| SS20 | 49170 | 27319 | 129939 | 30882 | 8533 | 86533 | 9307 | 82026 |
| SS23 | 52956 | 75954 | 108667 | 15323 | 7813 | 3406 | 10506 | 33491 |
| SS24 | 56510 | 38212 | 99756 | 12114 | 7131 | 20541 | 17808 | 33521 |
| SS27 | 61880 | 25383 | 121169 | 12530 | 4358 | 5431 | 50159 | 62241 |
| SS28 | 51758 | 20828 | 143259 | 20881 | 4781 | 1240 | 15177 | 64829 |
| SS35 | 41061 | 44661 | 88356 | 12501 | 6087 | 91850 | 17587 | 129544 |
| SS36 | 45679 | 88884 | 132334 | 4655 | 9513 | 142386 | 11970 | 50016 |
| SS37 | 56262 | 80953 | 150140 | 17624 | 6041 | 52565 | 27150 | 197544 |
| SS38 | 41113 | 33347 | 125096 | 23488 | 4830 | 32331 | 9998 | 72798 |
| SS39 | 65018 | 30470 | 186577 | 15250 | 4998 | 2078 | 30717 | 5807 |
| SS40 | 54929 | 91272 | 107202 | 26482 | 10772 | 76127 | 31557 | 80415 |
| Non-malignant CD4^+^ T cells (SS patients=21) | | | | | | | | |
| mean±  SD | 64708±5548 | 69797±10932 | 150311±16349 | 35419±9960 | 10680±4468 | 56137±36807 | 4828±  1890 | 12074±13821 |
| CD4^+^ T cells (HDs=10) | | | | | | | | |
| mean±  SD | 61722±6780 | 67154±11888 | 161890±12373 | 31344±9492 | 13454±3920 | 77626±36781 | 4255±  440 | 5005±  1260 |

**Table S6: Individual values of the median fluorescence intensity (MFI) of malignant SS cells and mean ± SD of non-malignant CD4^+^ T cell from SS patients (n=21) and CD4^+^ T cells from HDs (n=10) for the indicated markers.** Red and green values represent MFI data falling below or above the HD MFI range (defined as mean ± 1SD), respectively. Abbreviations: BV, Brilliant Violet; PacB, Pacific Blue; PE, Phycoerythrin; RB, RealBlue.

| SS ID | Genotype  rs10748643 | Genotype  rs7096317 | Survival  (months) | Status last follow-up | Kaplan-Meier analysis |
| --- | --- | --- | --- | --- | --- |
| SS02 | G/G | A/A | 66 | A | Y |
| SS06 | A/- | G/- | 143 | A | Y |
| SS07 | A/G | G/A | 125 | A | Y |
| SS08 | A/- | G/- | 54 | A | Y |
| SS09 | G/G | A/A | 62 | A | Y |
| SS10 | G/G | A/A | 98 | D | Y |
| SS12 | A/A | G/G | 39 | D | Y |
| SS14 | A/G | G/A | 37 | D | Y |
| SS15 | A/G | G/A | 35 | A |  |
| SS18 | A/G | G/A | 36 | D | Y |
| SS19 | A/A | G/G | 14 | D | Y |
| SS20 | A/G | G/A | 34 | D | Y |
| SS22 | A/A | G/G | 18 | A | Y |
| SS23 | A/G | G/A | 22 | A | Y |
| SS25 | A/G | G/A | 21 | A | Y |
| SS27 | G/- | A/- | 31 | A | Y |
| SS28 | G/G | A/A | 8 | A | Y |
| SS30 | A/G | G/A | 5 | A | Y |
| SS31 | A/A | G/G | 7 | A | Y |
| SS32 | A/G | G/A | 6 | A | Y |
| SS34 | A/G | G/A | 1 | D | Y |
| SS35 | G/- | NA | NA | D |  |
| SS36 | A/- | NA | NA | A |  |
| SS37 | A/- | NA | NA | A |  |
| SS38 | G/- | NA | NA | A |  |

**Table S7: Genotype distribution of rs10748643 and rs7096317 in our SS cohort (n=25), and patients analysed for survival analysis.** Abbreviations: A, alive; D, dead; Y, yes.
